# Similar contrast sensitivity functions measured using psychophysics and optokinetic nystagmus

**DOI:** 10.1038/srep34514

**Published:** 2016-10-04

**Authors:** Steven C. Dakin, Philip R. K. Turnbull

**Affiliations:** 1 School of Optometry & Vision Science, The University of Auckland, New Zealand

## Abstract

Although the contrast sensitivity function (CSF) is a particularly useful way of characterising functional vision, its measurement relies on observers making reliable perceptual reports. Such procedures can be challenging when testing children. Here we describe a system for measuring the CSF using an automated analysis of optokinetic nystagmus (OKN); an involuntary oscillatory eye movement made in response to drifting stimuli, here spatial-frequency (SF) band-pass noise. Quantifying the strength of OKN in the stimulus direction allows us to estimate contrast sensitivity across a range of SFs. We compared the CSFs of 30 observers with normal vision measured using both OKN and perceptual report. The approaches yield near-identical CSFs (mean *R* = 0.95) that capture subtle intra-observer variations in visual acuity and contrast sensitivity (both *R* = 0.84, *p* < 0.0001). Trial-by-trial analysis reveals high correlation between OKN and perceptual report, a signature of a common neural mechanism for determining stimulus direction. We also observe conditions where OKN and report are significantly *decorrelated* as a result of a minority of observers experiencing direction-reversals that are not reflected by OKN. We conclude that there are a wide range of stimulus conditions for which OKN can provide a valid alternative means of measuring of the CSF.

Visual acuity remains the gold standard for clinical assessment of vision despite contrast sensitivity being a better predictor of functional vision^1^. In the lab one measures the minimum contrast (threshold) supporting detection of a pattern at different spatial frequencies (SFs), to derive a psychophysical contrast sensitivity function ΨCSF^2^. Such a procedure is too slow to use in the clinic, and adaptive alternatives^3^,^4^ use Bayesian inference to determine the optimal combination of contrast and SF to present on a given trial. Although more efficient such techniques remain reliant on perceptual report, which is degraded by extraneous factors such as fatigue, compliance, attention^5^,^6^, feedback history^7^ or attribution of stimuli to non-causal events^8^.

Alternative, objective estimates of the CSF have been obtained with scalp-recordings of visual-evoked potentials (VEPs) that correlate well with psychophysical measurements at lower SFs (transient VEPs, *R*^2^ = 0.81 with dynamic stimuli^9^). Both pattern-reversal VEPs^10^,^11^ and “sweep” VEPs (that cycles through a set of SFs and contrasts) produce CSFs of similar shape to the ΨCSF, but with substantially lower sensitivity (e.g. 0.62–0.79 log units^12^).

Another route to the CSF infers participants’ ability to see the stimulus from their *eye movements*. Forced preferential looking (FPL) relies on children preferentially fixating patterned over uniform stimuli. Detection can be quantified by scoring eye movements either by a judge^13^, or an automated analysis of eye position^14^. However, estimates of visual function from FPL are necessarily lower bounds on performance, since the procedure cannot distinguish failures of detection from inattention. Alternatively, *involuntary* eye movements are less reliant on visual attention or compliance^15^ and have been used to probe visual function. The *ocular following response* OFR^16^; is a rapid tracking eye-movement, measured over short time periods (150–200 ms), made in response to sudden motion. OFR is mediated by cortical motion processing areas MT/MSTs^17^, although Sheliga *et al*.^18^ have shown that tuning of the OFR for SF and contrast can be captured by a non-direction-selective receptive field model, suggesting the OFR is *limited* by subcortical mechanisms.

Longer periods of unidirectional motion will induce a second class of involuntary eye-movement: *optokinetic nystagmus* (OKN). OKN is a conjugate oscillation of the eyes consisting of a slow tracking movement in the stimulus direction (where initial tracking corresponds to OFR), followed by a rapid restorative movement in the opposite direction (demonstration at https://www.youtube.com/watch?v=nVvZCl5M9nM). OKN serves to minimise “retinal slip” by partially stabilising images on the retina. Indeed, the reader may have noted this phenomenon when viewing the eye movements of a fellow train passenger who is looking out of the window. Young infants exhibit asymmetrical OKN (stronger temporal to nasal movements) - suggestive of a subcortical mechanism^19^ – that becomes more symmetrical by 24 months^20^. Like OFR, MT/MST is involved in generation of OKN e.g. lesions in these areas in macaques leads to inaccurate tracking during OKN^21^.

A considerable amount of work has examined the relationship between human OKN and functional vision. Correlations of Snellen acuity with the highest SF that elicits OKN, range between 0.6 and 0.85^22^,^23^,^24^, although see Cetinkaya *et al*.^25^. Here we revisit the problem of assessing the similarity of estimates of visual function measured with OKN and perceptual report. In order to minimise the impact of extraneous factors (e.g. motivation/attention, etc.) we made concurrent measurements of OKN and report. We assessed both the similarity of ΨCSF and oCSF and the extent to which these behavioural measures tap into the same neural mechanism. We conducted two experiments, the first to determine a procedure to make unbiased estimates of contrast sensitivity from OKN, the second to measure CSFs using OKN and report.

## Material and Methods

### Apparatus

Stimuli were presented in greyscale on a CRT monitor (22″ Sony Trinitron; 85 Hz; 1600 × 1200) driven by a video-processor (Bits#, Cambridge Research System, UK) controlled by a personal computer (the “Stimulus PC”), giving 14-bit contrast resolution. The display was viewed binocularly to minimise the contribution of any nasal/temporal asymmetries in optokinetic response to horizontal motion that may persist into adulthood^20^. Viewing distance was 72 cm to give a pixel density of 60 pixels per degree. Display luminance was gamma calibrated using a photometer (LS100, Konica Minolta, Japan). Experiments were written in Matlab (MathWorks, Natick, MA) using elements of the Psychtoolbox^26^. Monocular (left-eye) eye movements were recorded at 85 Hz using an Eyelink 1000 Infrared Eyetracker (SR Research, Ontario, Canada) in remote mode, allowing eye tracking without the use of a chin-rest. Eye movements were streamed to the Stimulus PC via a high-speed Ethernet connection and were read into Matlab using the Eyelink Toolbox^27^.

### Participants

In the first experiment we used a group of 6 volunteers with normal or corrected-to-normal vision (2 females, mean age 27.6 ± 10.1 years). In the second experiment this group was expanded to 30 participants (18 females, 26.3 ± 6.3 years). The experimental protocols and procedure were approved by the University of Auckland Human Research Ethics Committee. The protocols and procedure complied with the Declaration of Helsinki, and written informed consent was obtained from all participants prior to the experiment.

### Stimuli

**Figure 1:**
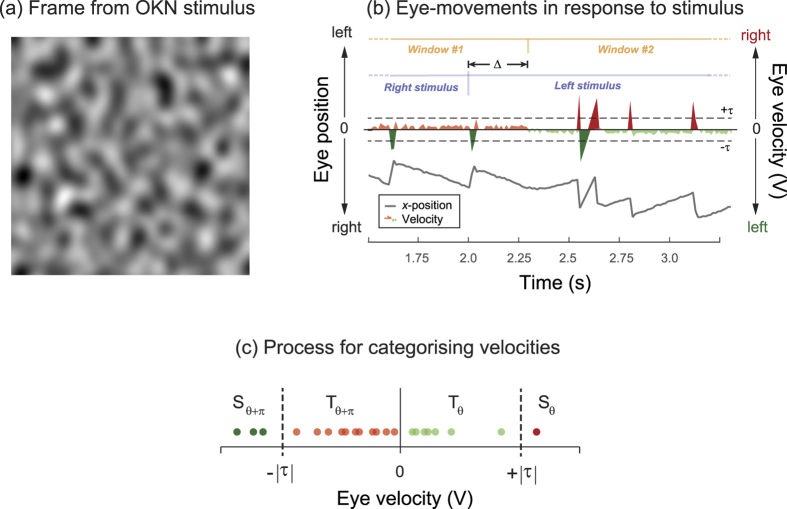
(**a**) A frame from the drifting stimulus used to drive OKN, (**b**) typical “sawtooth” pattern of eye positions (grey line) elicited by a rightward then a leftward drifting stimulus. The red/green shaded trace is the first derivative (velocity) coded for sign (green = left, red = right). Periods of tracking in the stimulus-direction interspersed by saccades in the opposite direction is characteristic of OKN. We allow for the latency in onset of OKN (here around 300 ms) by analysing a 2 second window of eye movements offset by a latency fixed per observer across all trials (Δ). (**c**) In order to categorise each instantaneous estimate of eye-velocity as either “saccade” or “tracking” we compare it to a threshold (τ), using its sign to categorise the direction (as θ or θ + π).

Stimuli were full-screen leftwards or rightwards drifting noise patterns. Specifically, patterns subtended 26.2 by 34.5 deg. Patterns were two-dimensional Gaussian noise (made with the randn command in Matlab), filtered to be isotropic and spatial-frequency (SF) band-pass (Fig. 1a). Filtering was performed in the Fourier domain using log-Gabor filters^28^. These filters are only specified in the Fourier domain but in the image domain (where they cannot be expressed in a closed form) they are “centre-surround” filters. Unlike other centre-surround filters (e.g. the Laplacian-of-Gaussian) these filters are constructed such that the range of SFs that they pass is constant (on log axes) as the peak SF changes. Contrast structure was flattened (to remove gross random fluctuations in contrast and so reduce trial-by-trial fluctuations in stimulus-energy) using a previously described technique^29^. The mean luminance of stimuli was 50 cd/m^2^. Stimuli had peak spatial frequencies of 0.94, 1.88, 3.75, 7.5 and 15 c/deg and a SF bandwidth of σ = 0.5 octaves. **Experiment 1** examined the effect of varying the velocity of full-contrast drifting noise patterns on the OKN signal strength, across SFs. Stimulus velocity was selected from the following values: 1.4, 2.8, 5.7, 11.3, 22.7, 45.3, 90.7, and 181.3 deg/sec. Stimuli were 170-frame (2-sec) movies played in a continuous sequence. Isotropic noise allows for measurement of OKN under higher speeds than gratings, while still retaining control of contrast/SF (unlike dot stimuli). **Experiment 2** examined the effect of SF and contrast on OKN using similar stimuli presented at a fixed velocity (10 deg/s) but with variable contrasts: either 0.2, 0.8, 1.6, 3.1, 6.3 12.5 or 25%.

### Procedure

Prior to data collection, we ran a 9-point calibration procedure on the eye tracker on each subject. In **Experiment 1** we presented 16 instances of every possible combination of the 5 SFs and 7 velocities to give a total of 560 movies within a single 18 m 40 s run. Stimulus SF, velocity and direction were randomised across trials; randomisation of direction and stimuli would minimise the build-up of perceptual or optokinetic aftereffects. Observers were instructed to view the stimuli and attempt to maintain central fixation (i.e. “stare”-OKN). Observers did not make a perceptual report. Eye movements were scored post-hoc for consistency with OKN, using the procedure described below (“*Measurement of OKN*”).

The procedure for **Experiment 2** was similar except (a) stimuli varied in contrast, not velocity and (b) observers made a continuous perceptual report of stimulus direction using the computer keyboard. We presented 16 instances of every possible combination of 5 SFs (same range as Expt 1) and 7 contrasts (0.2, 0.8, 1.6, 3.1, 6.3, 12.5 or 25%) to give a total of 560 movies within a single run. Observers performed a minimum of two runs. We occasionally ran observers with some additional low or high contrast conditions if their psychometric functions were at or near ceiling/floor with the contrasts as presented. With respect to the behavioural task, details of this procedure are given below (“*Analysis of behavioural data*”). Instructions were otherwise identical to Experiment 1 and again, stimulus order and direction was randomised across trials.

### Measurement of OKN

We developed an automated method for quantifying OKN from the output of the eye tracker (Fig. 1b). We start with the raw horizontal position of the eye (lower part of Fig. 1b, grey line); note the “sawtooth” pattern of “track-and –saccade” characteristic of OKN. We then compute the first derivative of x-position to give *V*, the horizontal velocity of the eye (Fig. 1b, red and green code right and left movement respectively). Note the switch from right-track/left-saccade to left-track/right-saccade and around 2.3 s, consistent with a switch from right to left OKN trailing the stimulus switch (at 2 s) by around 300 ms. We next classify eye movements as either saccades or tracking based on the magnitude of *V*. This classification process is illustrated in Fig. 1c for a series of estimates of *V* (filled circles). If we assign the stimulus direction (θ) a positive velocity then eye-velocities are classed as saccades (*S*) if their magnitude exceeds a “saccade-threshold” (*τ*; the first free parameter in our analysis), and are classed as tracking (*T*) if they do not. The sign of *V* indicates if a measurement is consistent with D_θ_ or D_θ+π_ In Fig. 1c, eye-movements consistent with OKN in direction θ (i.e. T_θ_ and S_θ+π_) are green, and for the opposite direction θ + π (T_θ+π_ and S_θ_) are red. To quantify OKN one can calculate D_θ_, the total distance travelled by the eye that was consistent with OKN in the direction θ:


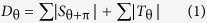


we can use this expression to evaluate D_θ_ and D_θ+π_ and then express D_θ_ as a proportion of total eye movement, to get the total OKN-like movement that is consistent with θ:


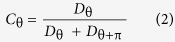


where C_θ_ varies between 0 (i.e. velocities entirely consistent with direction opposite to stimulus), +0.5 (i.e. random velocities) and +1 (i.e. velocities entirely consistent with stimulus). When using C_θ_ to predict trial-by-trial performance, we score values greater than 0.5 as correct and less than 0.5 as incorrect (with a value of 0.5 being scored randomly). Our analysis of eye movements must accommodate the known dynamic properties of OKN, in particular the latency that precedes its initiation, given that our stimuli consist of sequences of 2 second movies. This is illustrated in Fig. 1b where we see a 300 ms delay before OKN switches to reflect the change in stimulus direction. We compensated for this by having a second free parameter in our analysis (latency, Δ) that determines the delay in relation to stimulus onset before eye movements contribute to the OKN calculation. We analyse eye movements over a 2 s window commencing from this delayed start time. This effectively co-registers eye-movements with the stimulus in time before we make a final estimate of D_θ_ and C_θ_.

When analysing eye-movement data we estimated the optimal values for *τ*_sacc_ and Δ by maximising D_θ_ and C_θ_ (independently, in separate analyses) over all possible combinations of saccade thresholds (7, 11, 14, 28 or 43 deg/s) and latencies (0, 235, 471, 706, 941, 1176, 1412, 1647 or 1882 ms). These parameters were determined separately for each observer (optimising only C_θ_ in the main experiment), and fixed across their respective trials. In pilot experiments we noted that one of our observers exhibited reversed OKN, making saccades in the stimulus direction followed by smooth tracking in the opposite direction. This phenomenon is thought to arise from observers own (typically latent) nystagmus interacting with the stimulus-induced oculomotor response^30^. To accommodate such systematic bias, we ran our parameter fitting on all observers with Equations 1 and 2 both as presented, and with an exchange of θ with θ + π for all trials. This allows eye-movements that were consistent with either OKN, or reversed-OKN to be scored as consistent with direction θ. Note that whether optimisation used the original or reversed version was determined across the *whole data set* for each observer (i.e. our analysis did not allow for intermittent instances of reversed OKN within the same observer).

### Analysis of behavioural data

In Experiment 2 we had observers make a continuous report of the perceived direction of stimuli using two keys on the computer keyboard. Observer depressed (without looking) one key for leftwards, and another for rightwards, perceived motion. Observers were permitted to respond either by (a) continually depressing one key or the other, (b) periodically pressing a key to reflect their current percept, or (c) pressing a key to signal when the stimulus changed direction. When analysing these data we populated frame-by-frame instances of “no-perceptual report” with the last reported direction. For a given run this yields a stream of binary left/right responses which were analysed in manner analogous to the analysis of eye-position data described above (i.e. more than half of the responses needed to be consistent with the stimulus for the trial to scored “correct”). Specifically, we estimated the behavioural response by measuring the ratio of responses to a given direction, to the sum of all responses to both directions (akin to Equation 2). We maximised this estimate across the same range of a latencies to model the delay in observers’ responses. Similar to the eye movement analysis, this latency was modelled and fixed for each observer. This analysis had no other free parameters.

### Estimating detection performance

At the conclusion of a run within Experiment 2, eyetracking data were analysed by first deriving measures of OKN consistency over the run (as in Experiment 1), and the stream of behavioural data modelled in the manner described in the preceding section. Next the directions inferred from report and OKN were compared to the stimulus direction and scored as correct or incorrect. The resulting estimate of proportion correct was plot as a function of stimulus contrast and fit with a Weibull psychometric function:


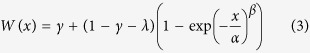


Fits were performed using Palamedes toolbox^31^, with a variable threshold (α), slope (β) and lapse-rate (λ), for a fixed rate of guessing (γ = 0.5). This yields an estimate of contrast threshold (α) - for report and OKN - for each SF presented. Fits were bootstrapped (using 1024 resamples) to derive confidence intervals on thresholds. We next plot contrast threshold as a function of spatial frequency ( *f* ) and fit the resulting CSF with a simple two-parameter model (using the iterative fitnlm routine in Matlab) of the form:


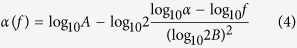


where *A* and *B* determine the height and slope of the CSF respectively. From this fit we can extrapolate acuity the SF leading to a sensitivity of 2.0^32^; and overall contrast sensitivity (the log of the area under the CSF). We bootstrapped these estimates by repeatedly resampling the bootstrapped threshold estimates (derived using the method described above), and refitting the model 1024 times. Acuity estimates were clipped in the range 1–45 c/deg to avoid outlier estimates made from degenerate bootstrapped data sets disproportionately influencing subsequent analysis.

## Results

### Experiment 1. Effect of velocity on OKN across different SFs

**Figure 2:**
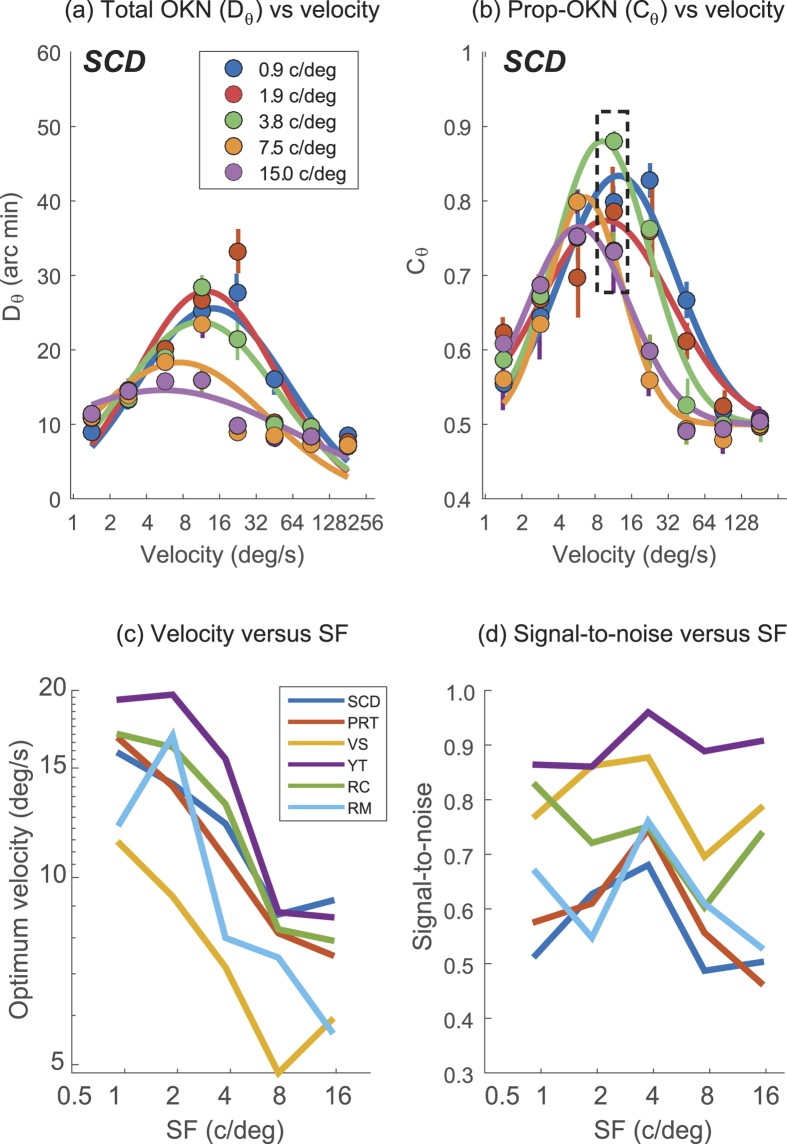
(**a**) The mean total distance moved by the eye of one observer that was consistent with OKN in the stimulus direction, for 16 drifting noise trials across five spatial frequencies, and eight velocities. Note substantially more OKN consistent motion at lower SFs. (**b**) As (**a**) but showing the total proportion of eye movements consistent with OKN in the stimulus direction (C_θ_). (**c**) The optimum velocity (derived from log-Gaussian fits to 6 observers similar to (**b**)) as a function of SF. (**d**) As for (**c**) but showing the amplitude of the log-Gaussian fit (a measure of signal-to-noise ratio) which does not show a strong dependence on SF, within observer.

Figure 2 plots our two measures of OKN (D_θ_ and C_θ_) – which have been optimised for this representative observer – as a function of stimulus velocity. Raw data have been fit with log-Gaussian functions. Note that the height of these functions scales with stimulus SF; higher spatial frequencies elicit weaker (i.e. lower amplitude) OKN. Note also that the peaks of these functions are shifted in relation to one another: at high SFs (e.g. green curves, 15 c/deg) most OKN is elicited at low velocities, while for low SFs (e.g. orange curve, 1.9 c/deg) more OKN is elicited at high velocities. These results accord with those of Schor & Narayan^33^ who measured “slow-phase gain” (SPG; the ratio of eye to target velocity in the tracking phase of OKN) in response to drifting gratings. SPG is proportional to *C* and *D* and these authors report (a) more OKN to lower SFs and (b) an inverse relationship between the maximum tolerable velocity and SF.

*C*_θ_ the proportion of eye movements that are consistent with OKN (Fig. 2b) shows a weaker dependence on SF than *D*_θ_ (the total distance travelled by the eye, consistent with OKN). This is manifest as curve amplitudes being more similar in Fig. 2b than 2a; note the highest amplitude curve in Fig. 2b is elicited by a mid-SF stimulus. Further, although the log-Gaussian fits to the data in Fig. 2a,b reveal a systematic shift towards higher velocities at lower SFs (summarised from C_θ_ across observers in Fig. 2c) it is clear that a single velocity of stimulus (≈10 deg/s; dashed box in Fig. 2c) elicits robust OKN across all observers and SFs.

Figure 2d summarises the amplitude of the log-Gaussian fit (a measure of signal-to-noise ratio; SNR) at this velocity, across SF for all observers. Note the reasonably consistent SNR across SF and within observer. This demonstrates that with a single velocity we can conduct testing across a wide range of SFs and be confident that a change in OKN signal is related to the stimulus, and not to a change in sensitivity of our measurement system. Pooled across all SFs and all observers, 10 deg/s maximised the total value of C_θ_ and this informed the velocity used for Experiment 2.

### Experiment 2: Comparison of contrast sensitivity measured using psychophysics and OKN

**Figure 3:**
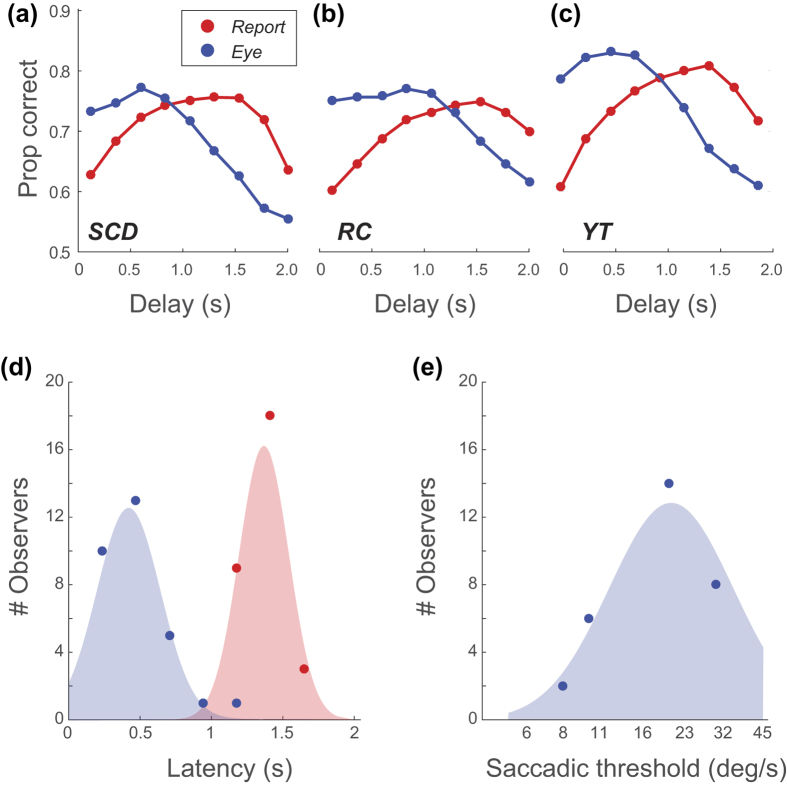
(**a**–**c**) Proportion of trials scored correct (pooled across all SF/contrast conditions) based on perceptual report (blue) or OKN (red), as a function of the latency applied to the analysis, for three observers. (**d**) Histogram of optimal latencies for report and OKN across all observers. (**e**) Histogram of saccadic thresholds.

Experiment 2 measured OKN (quantified using C_θ_) for stimuli of varying contrast and SF drifting at a velocity of 10 deg/sec. We first maximised the proportion of trials scored correct by our OKN analysis and report data across all conditions tested by optimising either one free parameter for behavioural data (latency) or two parameters for OKN data (latency and saccadic threshold). Figure 3a–c plots the trials scored as correct for different latency parameters, used to select the optimal latency across all trials for each subject, for both OKN and report. Results pooled across observers (Fig. 3d) indicate that a substantial latency on our OKN measure (typically around 500 ms), and a greater latency (nearer 1500 ms) on perceptual report, is required to optimise performance. Our OKN-latency parameter was set to values that are longer than typical latencies for OKN^34^. Similarly, our latency parameter for report is substantially longer than visual reaction times^35^. This is likely because our latency parameters accommodate not just delays in initiating a response (either OKN or report) but - because stimuli appeared within a continuous sequence - also delays in extinguishing the previous response. The report response latency is significantly delayed compared to the reflexive OKN, because it takes longer to reach its respective decision threshold^36^, and observers may have been pooling evidence before responding, driving the average latency up. With this said, our stimuli were presented in a continuous sequence, and we cannot rule out the latency parameter being linked to delays in processing arising from sequential trial effects. For example, rapid motion aftereffects are known to arise (under optimal conditions) after only 300–400 ms of adaptation^37^. However many aspects of our stimuli (variable/low contrast, random direction sequence, unambiguous direction of motion) should minimise the impact of any such interactions between stimuli.

**Figure 4:**
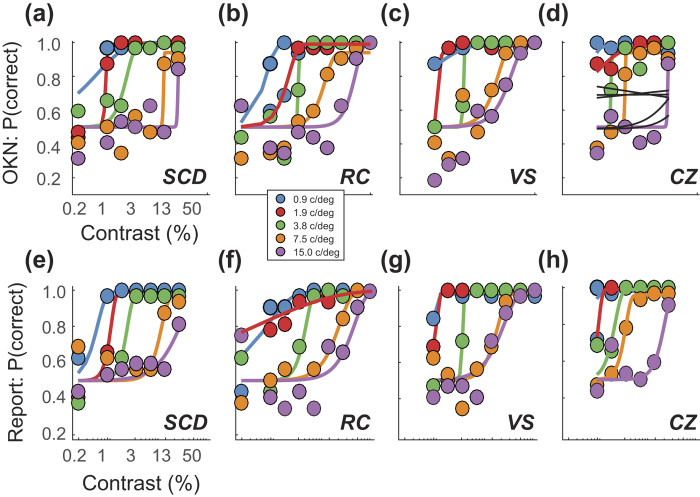
Proportion of correct reports of stimulus direction based on (**a**–**d**) our OKN analysis and (**e**–**h**) perceptual report. Data are shown for four observers tested with five spatial frequencies. Fits (coloured lines) based on a Weibull function provide a good account of the data. Note similarity of fits across conditions, with one notable exception at 1.9 c/deg in Fig. 4b,f, where the fits differ due to the low contrast conditions. Observer CZ (Fig. 4d) exhibited reversed OKN and our analysis took this into account. Had it not, performance would have been substantially poorer (fits shown by black lines).

We compared the proportion of trials scored correct based on OKN consistency (C_θ_) to one based on perceptual report, pooled over trials within each contrast and SF combination. Figure 4 plots the proportion of trials scored correct as a function of stimulus contrast, based on (a–d) our measure of C_θ_ and on (e–h) observers’ perceptual report. Data have been fit with Weibull functions, which provide a good account of the data. Note that both measures vary consistently across SF, demonstrating that the oCSF can be a sensitive candidate-measure of the ΨCSF.

**Figure 5:**
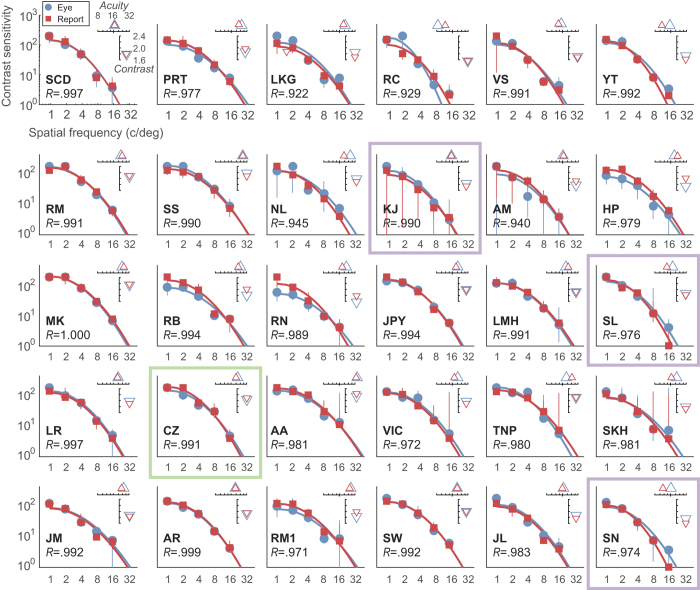
Contrast sensitivity for drifting noise measured for thirty observers using perceptual report (red) and OKN (blue). The plots show thresholds derived from the Weibull fits to raw data (Fig. 3), error bars show ±1 SD based on a bootstrap of the fit, and the solid coloured lines are the fits of a two-parameter model (Equation 3). The open triangles plot estimated acuity (Δ) (the SF at which the contrast sensitivity equals 2) and overall contrast sensitivity (∇) (the log_10_ of the area under the fit curve). Note the high levels of agreement between data and between data and fits. All correlations between each observer’s ΨCSF and oCSF were very strong (*R* > 0.92) and significant at less than p < 0.05. The green box indicates data from one observer who showed reversed OKN, and the purple boxes show data where observers exhibited trial-by-trial disagreement between OKN and report (see *Trial-by-trial agreement*, below).

We next extract the scale-parameter (α) from the Weibull fits, as an estimate of threshold, across all SFs to derive contrast sensitivity functions (the threshold contrast levels supporting reliable identification of stimulus direction). CSFs for all 30 observers are plotted in Fig. 5 with oCSFs shown in blue and ΨCSF in red. Fits are from the two-parameter model defined in Equation 3. For each observer we observe significant correlation between thresholds measured using OKN and report (*R* values are given in Fig. 5). For data showing weaker agreement (e.g. observer RC on the top row on Fig. 5), examination of raw data (Fig. 4b,f) reveal that the discrepancy arises from data measured at a single SF (1.9 c/deg; shown in blue in Fig. 4). Here the difference in fit is driven by only a single pair of data points (i.e. 2 × 16 trials) at the lowest contrast level. This arose from our use of a method of constant stimuli with a single range of contrasts for all SFs, which occasionally led to the fitting of our psychometric functions being under-constrained.

Figure 5 plots estimated acuity (embedded x-axis; Δ symbols) and overall contrast sensitivity (embedded y-axis; ∇ symbols) derived from both OKN (blue) and report (red). Visual acuity is defined as the SF leading to a contrast sensitivity of 2.0^38^. The contrast value is defined as the log of the area under the fit function^2^, computed in analogy with Pelli-Robson contrast charts^39^. All observers’ oCSF and ΨCSF were significantly and very highly correlated (mean *R* = 0.95, minimum *R* = 0.922, all p < 0.03).

Above we noted that one of our observers (CZ, Fig. 4d,h and framed in green in Fig. 5) consistently exhibited *reversed OKN*, making saccades in the stimulus direction followed by tracking in the opposite direction^30^. Our analysis accommodates this difference by taking the maximum C_θ_ across all of each subjects’ trials computed using the true stimulus direction (θ), or with the opposite direction θ + π. With the stimulus direction inversion, CZ’s oCSF is in the normal range and agrees closely with their ΨCSF.

**Figure 6:**
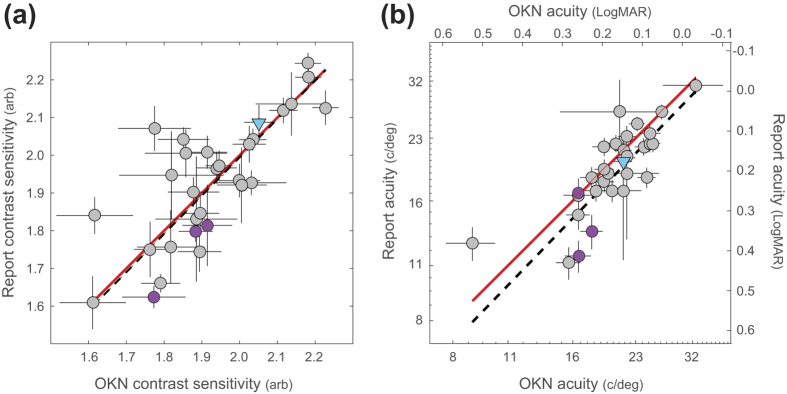
Correlations between the OKN-based measures and perceptual report of (**a**) Contrast sensitivity and (**b**) visual acuity. Note that the range in contrast sensitivity estimates spans approximately half an octave, while 29 of the visual acuity estimates span a full octave (1 observer appeared to have much poorer visual acuity on both measures). The dashed lines represent the error-weighted correlations between each measure (Both *R* = 0.84, p < 0.0001), while the red lines demonstrate agreement. Data from 30 observers are shown, error bars indicate ±1σ of the estimates derived from bootstrapping the CSF fits. Purple symbols indicate observers who showed significant disagreement between OKN and report, and blue symbols indicate an observer who exhibited reversed OKN.

Although we tested observers with ostensibly normal vision, we note considerable inter-observer variation in acuity and contrast sensitivity. This is highlighted in Fig. 6a,b which plots the contrast sensitivity and acuity estimates for each individual, calculated from the oCSF and ΨCSF shown in Fig. 5. Across individuals, there is a high level of agreement between OKN and report-based measures. A correlation (weighted by the elliptical error for each datum) yields a value of *R* = 0.84, p < 0.0001 for both the visual acuity and contrast sensitivity data. A least-squares fit of a line to the data (dashed line, Fig. 6a) shows that OKN-measures give an unbiased estimate of perceptual contrast sensitivity, with a small (~1.3%) systematic bias which underestimates reported acuity (dashed line, Fig. 6b).

### Trial-by-trial agreement

**Figure 7:**
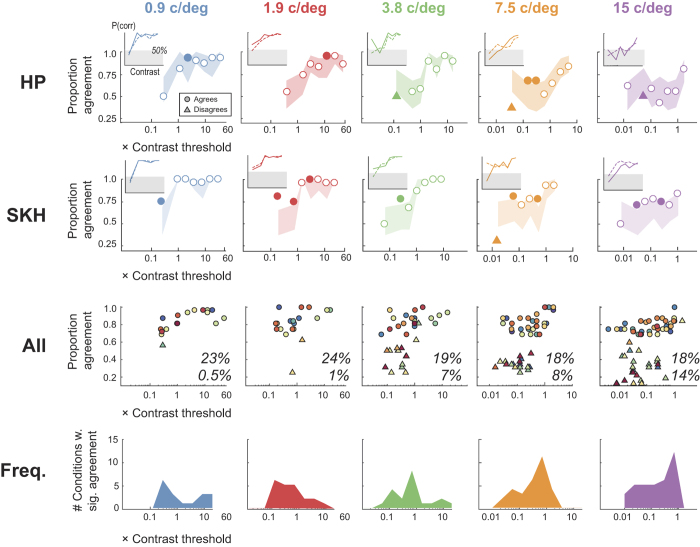
(Rows 1 and 2) Average trial-by-trial agreement of direction estimates based on report and OKN for two observers. Each graph plots agreement as a function of stimulus contrast (as a multiple of each observer’s threshold) for a single SF. The range of agreement expected by chance is the shaded region, with agreement shown as open symbols, and significantly (p < 0.05) higher and lower levels of agreement plot as filled circles and triangles, respectively. Inset in each figure are the psychometric functions for (solid line) report and (dashed line) OKN; shaded region indicates performance falling below chance. (**Row 3**) Points of significant agreement and disagreement for all 30 observers; the inset numbers are the total percentage of agreement (top) and disagreement (bottom) estimates that were significant out of the number that could have been significant. Note a clustering of disagreements around lower contrast, high SF conditions. (**Row 4**) Histograms of number of conditions leading to significant agreement across all observers. Note peaks around 0.75× threshold indicating that this level of uncertainty is optimal for assessing agreement.

Our data allow us to go a step further and examine the trial-by-trial consistency – across both OKN and key responses - for each combination of SF and contrast. For a given sequence of trials, we can calculate the % agreement between OKN and report (across a set of - typically 32 -trials with the same SF and contrast) and use binomial statistics to estimate the probability that this level of agreement arose by chance (two tailed test, α = 0.05 level). Figure 7 summarises the results of this analysis of data from Experiment 2 (n = 30 observers, 34320 total trials analysed). Note that for conditions leading to high levels of performance (e.g. high contrast stimuli) the upper confidence limit on the expected level of agreement includes perfect agreement, so we cannot assign a probability of exceeding this range. The same is true of the lower confidence interval if it exceeds zero agreement. Under either of these conditions it is not possible for us to show a level of agreement that differs from what we would expect by chance. Therefore, having counted the instances of statistically significant agreement we express this as a proportion of all conditions (where condition is a given combination of contrast and SF for each observer) that *could have* shown significant agreement (634 out of 1070 conditions). Responses in 19.7% of such conditions were more correlated than chance would predict. This extra level of agreement observed in a significant proportion of our OKN and key press data is strongly suggestive that both measures are tapping into a common neural resource that encodes stimulus direction. We note that (voluntary) smooth pursuit also shows trial-by-trial agreement with perceptual report^40^.

However, note that as spatial frequency increases, the number of *disagreements* also increases; at 15 c/deg, 14% of trials were associated with significant levels of disagreement across measures. Such disagreements are skewed towards low-contrast stimuli. We further note that 38% (24/64) of disagreements originate from three observers whose data are identified using purple symbols in Fig. 6, with lower contrast sensitivity (all 3 observers) and acuity (2/3 observers) estimates. Examining these individuals’ CSFs in Fig. 5 (purple boxes), reveals that SL and SN show very poor sensitivity for 15 c/deg stimuli based on their perceptual report (but not on their OKN). The psychometric functions from these observers – measured with high spatial frequency, low contrast stimuli – are consistent with their experiencing *reversal of perceived direction*. This in turn leads to higher estimates of threshold under these conditions. Examination of individual psychometric functions from other observers (e.g. Figs 4f and 7, insets for 7.5 and 15 c/deg) confirms a general trend for disagreements to result from perceptual report (solid line) falling below 50% chance level at high SFs/low contrasts, while the OKN function reduces to chance as expected. These data indicate that for some observers, under certain conditions, there is a dissociation between visual processing motion supporting perceptual report and OKN. We consider the implications of this in the Discussion.

### Bias

**Figure 8:**
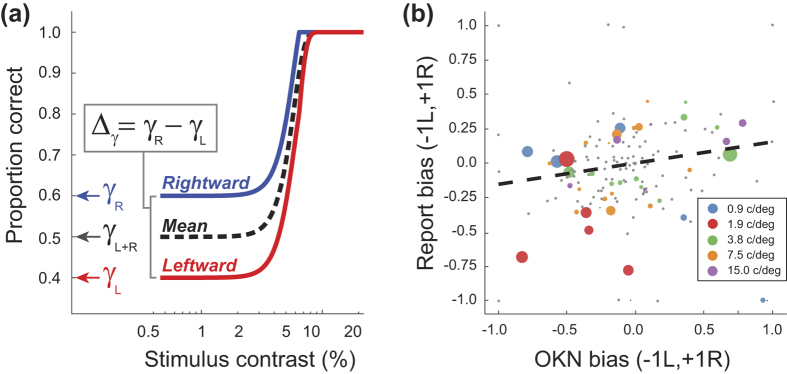
(**a**) Schematic psychometric functions based on responses to rightward (blue) and leftward (red) motion, or responses over all stimuli (dashed-line). A bias towards responding to the right will lead to more correct responses for rightward than leftward stimuli, even with stimuli that are sufficiently low contrast to be effectively invisible. This behaviour is captured by the γ parameter of a Weibull function. The difference in γ, across data collected with left and right stimuli, quantifies bias as a value between −1 (strong bias to leftwards response) and +1 (strong bias to rightwards response) with 0 corresponding to unbiased behaviour. (**b**) Estimated bias (Δγ) for OKN and report. Grey and coloured symbols are levels of bias that either were or were not significantly greater than 0. The fit is based on a linear regression (inversely weighted by the confidence intervals on the bias estimates; weights determine symbol size) and shows a significant correlation of response bias across OKN and report.

Our experiment required that observers identify if a stimulus was moving left or right, which necessitates their use of a *criterion*: the level of evidence supporting a switch from reporting left to reporting right (or vice versa). Consequently, our analysis of both OKN and report data is vulnerable to *response bias* – a systematic tendency (for report or OKN) to favour one direction over another. Figure 8a illustrates how such bias could impact on behaviour. A tendency to favour reporting to the right will lead to superior performance for rightward stimuli regardless of their contrast (i.e. even when the observer should be guessing), and correspondingly poorer performance for leftward stimuli. This tendency is captured by the γ (guess rate) parameter of the Weibull (Equation 3) which sets the performance level at which the psychometric function plateaus. When previously fitting data we assumed this value to be 0.5 (allowing us to fix this value, and reduce the number of free parameters on our fit from four to three).

We derived our bias estimates by splitting our identification data into two sets (responses to stimuli moving rightwards or leftwards), and fitting each with a Weibull function (red and blue lines, Fig. 8a). This fitting was as described, except that while we fit β (slope), λ (lapse-rate) as before, we now fit γ instead of threshold, α (which we set to be the value from the previous analysis). From pairs of estimates of γ across the two sets (γ_*L*_ and γ_*R*_ we could derive the signed difference in bias across L/R stimuli (Δ_*γ*_) for both report and OKN data. As well as collapsing pairs of guess-rates into a single bias estimate (−1 to +1, for bias towards left and right responses, respectively) this has the advantage of leveraging statistical power from all of our data. By bootstrapping the fits (using the resampling method described) we could compute distributions of Δ_*γ*_ to determine if such estimates were significantly different from the no-bias value of 0 (with a significance level set to be 0.001, a conservative approximation to the Bonferroni corrected significance level of p = 0.05/150, necessitated by our use of 1024 bootstraps).

Figure 8b plots 150 estimates (30 observers ×5 conditions) of response-bias estimated from OKN and report. Grey symbols (69% of data) are conditions where Δ_*γ*_ was not significantly different from 0 for either report or OKN, and coloured symbols are conditions with significant bias. Symbol size codes our confidence in estimated bias (the inverse of the mean variance across the OKN- and report-based estimates). This confidence was used to weight a linear regression (run only on Δ_*γ*_ pairs with significant bias; *n* = 44) which yielded a slope of 0.154 (R^2^ = 0.09, *p* = 0.048). Thus, there is a significant (albeit modest) correlation in response-bias across report and OKN. We attribute this dependence on fluctuations in the motion signal-strength in our noise patterns that are influencing the response of a common motion processor. However, we cannot rule out the influence of attention on bias (which could in turn manifest as a top-down influence on OKN.) That this is a weak dependence is likely due to response bias estimates being contaminated by the effect of other performance limits (e.g. lapse-rate, inter-trial effects).

## Discussion

We have described an automated method for measuring a contrast sensitivity function (CSF) using an automated analysis of optokinetic nystagmus (OKN) and compared the results to a CSF obtained using conventional perceptual report (ΨCSF). Our results indicate a high level of agreement between the two methods, with both capturing subtle variations in acuity and overall contrast sensitivity in observers with ostensibly normal vision. Further, trial-by-trial agreement between these methods suggests the oCSF and ΨCSF rely (over a wide range of conditions) on a common neural mechanism for signalling motion. We contend that our results validate the use of OKN as a proxy for standard psychophysical assessment of acuity and contrast sensitivity.

While our work indicates a close link between OKN and functional vision, there are several challenges in translating our methodology into a clinical vision test. For example, Schor and Levi^41^ examined OKN in amblyopia and reported that although their oCSF was similar to their ΨCSF for nasal motion, it was substantially lower (≈1 log unit) when measured with temporal motion. Here we avoided this issue by using binocular presentation (providing both nasal and temporal motion) which should neutralise the impact of any functional asymmetry. However, such asymmetries are relevant when we consider the feasibility of *monocular* tests of OKN (necessary for it to be clinically useful). From a methodological standpoint we note that Schor and Levi obtained their contrast detection thrtesholds using two different methods of adjustment based either on the observers’ report or on whether the eye movement trace met was judged to have met the criterion for OKN (5 cycles over 20 s). The different criteria used for each observers’ oCSF and ΨCSF, makes it difficult to interpret *absolute* differences between measures (although one can legitimately compare *relative* performance). It remains to be seen if our own methodology – which is less dependent on the amplitude or frequency of OKN – would produce a similar discrepancy.

We note that one of our observers exhibited reversed OKN^30^,^42^. Because our analysis accommodated this we were able to obtain excellent agreement between OKN and report-based estimates of contrast sensitivity and acuity. The cause of this phenomenon is thought to be an interaction between a latent nystagmus and OKN, and our results shed light on the mechanism leading to reversal of OKN. Gresty *et al*.^43^ propose two possible mechanisms: either poor cortical motion processing, or masking of OKN by the mechanism responsible for latent nystagmus. Given that our observer exhibited normal perceptual report in response to stimuli that led to reversal of their OKN our data support the latter hypothesis. This is not the case for overt nystagmus, e.g. associated with albinism, which leads to a behavioural deficit in the processing of both local and global motion^44^. We do not yet know if the proposed method can provide an accurate assessment of cortical motion processing in the presence of overt oculomotor abnormalities such as nystagmus.

Significant trial-by-trial disagreement arose (typically at high SFs/low contrasts) for several observers. Examination of their psychometric functions often revealed that while performance based on OKN fell close to chance, performance based on report had fallen *below* chance, indicating that that perceived direction was opposite to stimulus direction. Note that we used successful identification of stimulus direction (leftwards versus rightwards) as a proxy for successful detection (present versus absent) to minimise impact of criterion-shift. So, for the conditions described above, while observers could often detect drifting stimuli, their reported experience of reversed motion led to very poor (below chance) identification and to an effective under-estimation of their detection performance. Unlike report, OKN performance was not anti-correlated with stimulus direction under these conditions, and this led to better performance. Our approach - like many of studies of contrast sensitivity - relies on identification. In this context OKN is a *better* reflection of the observers’ inability to signal the correct direction than perceptual report, while being a poorer reflection of the observer’ visual experience. This is borne out by that fact that 2/3 of observers who showed high levels of OKN-report disagreement also showed abnormally low acuity and contrast sensitivity (based on report but not OKN; Fig. 6).

The mismatch between report and OKN however, does indicate that, despite a significant overlap between the neural mechanisms supporting each, certain conditions can engage distinct motion processing. This is consistent with earlier studies describing (for example) a weak OKN response to second-order motion SOM^45^; but which can modify the response to simultaneously presented first-order motion^46^. We speculate that different response to low-contrast/high-SF drifting patterns by OKN arises from reduced sensitivity to SOM in these patterns. That SOM can influence OKN^46^ implicates the cortical areas supporting SOM notably areas V3 and VP^47^; in OKN. A variety of other evidence supports a central role for MT in OKN, e.g. bi-directional motion reduces the slow-phase OKN-response when compared to unidirectional motion^48^, which mirrors the reduced responsivity of motion-selective cells in MT but not V1^49^.

We note that OKN has been traditionally divided into two types based on the instructions given to an observer: ‘stare’ (i.e. attempt to fixate) and ‘look’ (i.e. follow the stimulus)^50^. In line with most previous studies, we used a ‘stare’ paradigm. It has been suggested that ‘look’ OKN invokes more higher-level processing^51^,^52^ although this could simply reflect the additional attention required to track a target^15^,^53^,^54^.

Here we used a method of constant stimuli to pre-select stimulus levels at the beginning of a run. This procedure is inefficient and requires many trials (around 1100 per observer). We did this because an adaptive procedure would have to be driven by either the OKN or report, and we did not wish to introduce differences from presenting one technique but not the other with an optimal stimulus range. Since establishing the feasibility of our approach, we have implemented an adaptive procedure that uses OKN to update a trial-by-trial estimate of the underlying CSF using the FAST toolbox^4^. Using the inferred CSF allows the procedure to select the most informative combination of SF and contrast to present on the next trial^3^,^4^. Such procedures are highly efficient, and reduce the number of trials required by a factor of 5–10 compared to a standard run of individual adaptive staircases^3^,^32^. We have run pilot experiments on children who viewed commercial/animated cartoons that were periodically replaced with drifting stimuli similar to those described above. Using a similar eyetracking and classification procedure, we were able to estimate the CSF of children as young at 28 months in around 7 minutes, using a procedure that did not require the child do anything except watch television.

## Additional Information

**How to cite this article**: Dakin, S. C. and Turnbull, P. R. K. Similar contrast sensitivity functions measured using psychophysics and optokinetic nystagmus. *Sci. Rep*. **6**, 34514; doi: 10.1038/srep34514 (2016).
